# Multiple amino acid changes are responsible for the shift of tuning breadth along the evolutionary trajectory of a moth pheromone receptor

**DOI:** 10.17912/micropub.biology.001075

**Published:** 2024-02-08

**Authors:** Zibo Li, Rémi Capoduro, Marie-Christine François, Emmanuelle Jacquin-Joly, Nicolas Montagné, Camille Meslin

**Affiliations:** 1 Institut d’Ecologie et des Sciences de l’Environnement de Paris (iEES-Paris), France, Sorbonne Université, INRAE, CNRS, IRD, UPEC, Université Paris Cité

## Abstract

Sex pheromone recognition is essential for mating in many insects and plays a major role in maintaining reproductive barriers. A previous study from our lab reported the evolutionary history of the pheromone receptor OR5 in *Spodoptera* moths. Using heterologous expression in *Xenopus* oocytes and site-directed mutagenesis, we found that eight amino acid substitutions were sufficient to recapitulate the evolution from an ancestral broadly-tuned to a highly specific receptor. Here, we confirmed this result using expression in *Drosophila* olfactory neurons. This further confirmed that multiple amino acid changes explain the shift in tuning breadth of *Spodoptera* OR5 during evolution.

**Figure 1. Multiple amino acid changes are responsible for the shift of tuning breadth of a moth ancestral pheromone receptor:**
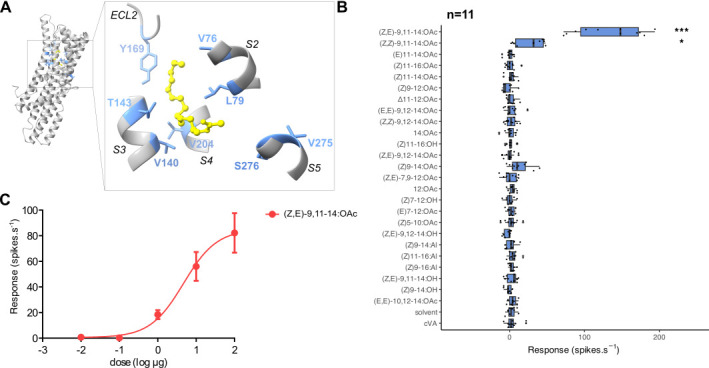
**A** . 3D model of AncOR5_75_mut8x and its predicted binding pocket. The 8 candidate amino acids are indicated in blue. S2, 3, 4 and 5: transmembrane helices, ECL2: extracellular loop 2. The pheromone compound ( *Z,E* )-9,11-14:OAc is indicated in yellow. **B** . Action potential frequency recorded in *Drosophila* at1 OSNs expressing AncOR5_75_mut8x after stimulation with 25 pheromone compounds (10 µg loaded in the stimulus cartridge). ***: corrected p-value<0.001, *: corrected p-value<0.05, Kruskal-Wallis rank sum test followed by a Wilcoxon pairwise test with a Benjamini-Hochberg correction for multiple testing. **C.** Dose-response curve of at1 OSNs expressing AncOR5_75_mut8x to different doses of ( *Z,E* )-9,11-14:OAc.

## Description

At the interface between the insect and its environment, olfaction is crucial for reproduction and host selection, and plays an essential role in the adaptation of species to their environment as well as in the speciation process. Insect odorant receptors (ORs) are seven transmembrane domain proteins that detect odorants, with the N-terminus being intracellular and the C-terminus being in the cytosol (Benton et al. 2006) . ORs are expressed at the membrane of the olfactory sensory neurons (OSNs) that are hosted within sensilla of the olfactory organs, mainly the antennae. They follow the classical birth-and-death model of evolution of multigene families (Nei et Rooney 2005) . High rates of tandem duplications are ultimately leading to functional divergence and the emergence of novel response spectra, as well as loss of genes by deletion or pseudogenization events (Sanchez-Gracia et al. 2009). Each species is thus evolving its own OR repertoire. We previously identified a couple of paralogous ORs in the moths *Spodoptera littoralis* and *Spodoptera litura* with divergent tuning breadths. In these two sister species, the major component of the sex pheromone is ( *Z,E* )-9,11-tetradecadienyl acetate (hereafter referred to as ( *Z,E* )-9,11-14:OAc). While OR5 is able to bind specifically ( *Z,E* )-9,11-14:OAc in both species, its duplicate OR75 is also a pheromone receptor but exhibits a broader tuning breadth (Li et al. 2023) . Detection of ( *Z,E* )-9,11-14:OAc is necessary and sufficient to trigger male attraction towards females in *S. littoralis* and *litura* . However, this compound is not found in the pheromone blend of other species of the *Spodoptera* genus, meaning that a major change occurred in the pheromone communication system of a common ancestor of these two species. To identify precisely what changes occurred at the receptor level, we used in this previous work an approach of ancestral gene resurrection and reconstructed the evolutionary trajectory of OR5 and OR75 by studying three different ancestral receptors: AncOR5, AncOR75 (post-duplication) and AncOR5_75 (pre-duplication). These ancestral ORs were resurrected through heterologous expression in *Drosophila* OSNs and we demonstrated that the specificity likely evolved after duplication only in the OR5 lineage while OR75 remained broadly tuned. More, a combination of 3D modeling of AncOR5_75 and *in silico* docking allowed to identify 28 amino acid positions potentially interacting with ( *Z,E* )-9,11-14:OAc, of which eight diverged between AncOR5_75 and AncOR5 (panel A of the figure). To verify whether these eight mutations could have been responsible for the emergence of specificity towards ( *Z,E* )-9,11-14:OAc, we compared response spectra of AncOR5_75 receptors carrying or not these 8 mutations, using *in vitro* expression in *Xenopus* oocytes and stimulation with a panel of six pheromone compounds. By doing so, we could demonstrate that the ancestral receptor carrying the mutations (hereafter referred to as AncOR5_75_mut8x) exhibited the same specificity as AncOR5, suggesting that multiple amino acid changes occurred during evolution to enable the shift of tuning breadth (Li et al. 2023) .

Here, we have endeavored to confirm the shift of specificity of AncOR5_75_mut8x observed in the *Xenopus* oocyte system by using another expression system and a larger panel of pheromone stimuli. To achieve our goal, we generated a *D. melanogaster* line expressing AncOR5_75_mut8x in at1 OSNs in place of the endogenous receptor DmelOR67d. These OSNs were then stimulated with air puffs odorized with a large panel of 25 pheromone compounds, and responses were monitored by single-sensillum recordings. ( *Z* , *E* )-9,11-14:OAc and its trans isomer ( *Z* , *Z* )-9,11-14:OAc significantly activated AncOR5_75_mut8x-expressing OSNs, with a mean response around 130 spikes.s ^−1^ and 28 spikes.s ^-1^ , respectively (panel B of the figure). Such a small response to ( *Z* , *Z* )-9,11-14:OAc is not unusual as it was also observed for SlituOR5 in the same heterologous expression system and for AncOR5 as well, although the response was not significant for the latter. Additionally, modest non-significant responses were recorded for ( *Z* )9-14:OAc. Dose–response analyses revealed that AncOR5_75_mut8x had similar detection thresholds to AncOR5, SlitOR5, and SlituOR5 (Li et al. 2023) . Altogether, these results confirm the specificity of AncOR5_75_mut8x towards ( *Z* , *E* )-9,11-14:OAc, which has now been validated in two distinct heterologous expression systems. Compared to the broad tuning breadth that was observed for the non-mutated AncOR5_75 in the same *Drosophila* OSN setting (Li et al. 2023) , it confirms that the simultaneous change of these 8 amino acid positions lead to a narrowly tuned pheromone receptor and further supports the hypothesis that multiple amino acid changes have been necessary for the alteration of the OR5 tuning breadth as well. This result confirms again the reliability of using *Xenopus* oocytes and two-electrode voltage clamp as a tool to deorphanize insect odorant receptors (Wang et al. 2016; Hou et al. 2019; Yuvaraj et al. 2021) .

## Methods

The AncOR5_75_mut8x mutant full-length open reading frame was optimized for expression in *Drosophila* , synthetized *in vitro* and subcloned into the pUAST.attb vector by Synbio Technologies (Monmouth Junction, NJ, USA). The transformant *UAS-AncOR5_75_mut8x* transformant line was generated by BestGene Inc. (Chino Hills, CA, USA) by injecting the pUAST.attB-AncOR5_75_mut8x plasmid into fly embryos with the genotype y1 M{vas-int.Dm}ZH-2A w*; M{3xP3-RFP.attP}ZH-51C (Bischof et al. 2007) . The *UAS-AncOR5_75_mut8x* balanced line was then crossed to the *Or67d* ^GAL4^ line (Kurtovic, Widmer, et Dickson 2007) to obtain double homozygous flies expressing AncOR5_75_mut8x in at1 OSNs instead of Or67d. Flies were reared on standard cornmeal-yeast-agar medium and kept in a climate- and light-controlled environment (25°C, 12h:12h light:dark cycle).

Twenty-five different pheromone compounds were used to screen the responses of at1 OSNs by using single-sensillum extracellular recordings, as previously described (de Fouchier et al. 2015) . The pheromone compounds were either synthesized in the lab or purchased from Sigma-Aldrich (St Louis, MO, USA) and Pherobank (Wijk bij Duurstede, The Netherlands) and were diluted in hexane (Carlo Erba Reagents, Val de Reuil, France). at1 OSNs were stimulated during 500 ms, using stimulus cartridges made of Pasteur pipettes containing 10 µg of pheromone dropped onto a filter paper. Dose-response analyses were performed using the same methods, with doses ranging from 10 ng to 100 µg of pheromone in the stimulus cartridge. Data were analyzed using GraphPad Prism 8 (GraphPad Software Inc., San Diego, CA, USA) and R. Odorants were considered as active if the response they elicited was statistically different from the response elicited by the solvent alone (Kruskal-Wallis followed by a pairwise Wilcoxon test with a Benjamini-Hochberg correction for multiple testing).

## Reagents

**Table:** 

**Strain**	**Genotype**	**Source**
*UAS-AncOR5_75_mut8x*	* yw; UAS-AncOR5_75_mut8x, w ^+^ ; + *	BestGene Inc.
*Or67d* ^GAL4^	* w ^+^ ; +; Or67d ^GAL4^*	Dickson lab
**Plasmid**	**Description**	**Source**
pUAST.attb -AncOR5_75_mut8x	Plasmid encoding for the ancestral OR carrying eight mutations	Synbio Technologies
